# Impact of a structured ICU training programme in resource-limited settings in Asia

**DOI:** 10.1371/journal.pone.0173483

**Published:** 2017-03-14

**Authors:** Rashan Haniffa, Yoel Lubell, Ben S. Cooper, Sanjib Mohanty, Shamsul Alam, Arjun Karki, Rajya Pattnaik, Ahmed Maswood, R. Haque, Raju Pangeni, Marcus J. Schultz, Arjen M. Dondorp, for the ICU Training in South Asia Group

**Affiliations:** 1 Centre for Tropical Medicine, Nuffield Department of Medicine, University of Oxford, Oxford, United Kingdom; 2 Faculty of Tropical Medicine, Mahidol University, Bangkok, Thailand; 3 Ispat General Hospital, Rourkela, Orissa, India; 4 Chittagong Medical College Hospital, Chittagong, Bangladesh; 5 Patan Hospital, Patan, Kathmandu, Nepal; 6 Department of Intensive Care, Academic Medical Centre, Amsterdam, the Netherlands; Nanjing University Medical School Affiliated Nanjing Drum Tower Hospital, CHINA

## Abstract

**Objective:**

To assess the impact on ICU performance of a modular training program in three resource-limited general adult ICUs in India, Bangladesh, and Nepal.

**Method:**

A modular ICU training programme was evaluated using performance indicators from June 2009 to June 2012 using an interrupted time series design with an 8 to 15 month pre-intervention and 18 to 24 month post-intervention period. ICU physicians and nurses trained in Europe and the USA provided training for ICU doctors and nurses. The training program consisted of six modules on basic intensive care practices of 2–3 weeks each over 20 months. The performance indicators consisting of ICU mortality, time to ICU discharge, rate at which patients were discharged alive from the ICU, discontinuation of mechanical ventilation or vasoactive drugs and duration of antibiotic use were extracted. Stepwise changes and changes in trends associated with the intervention were analysed.

**Results:**

Pre-Training ICU mortality in Rourkela (India), and Patan (Nepal) Chittagong (Bangladesh), was 28%, 41% and 62%, respectively, compared to 30%, 18% and 51% post-intervention. The intervention was associated with a stepwise reduction in cumulative incidence of in-ICU mortality in Chittagong (adjusted subdistribution hazard ratio [aSHR] (95% CI): 0.62 (0.40, 0.97), p = 0.03) and Patan (aSHR 0.16 (0.06, 0.41), p<0.001), but not in Rourkela (aSHR: 1.17 (0.75, 1.82), p = 0.49). The intervention was associated with earlier discontinuation of vasoactive drugs at Rourkela (adjusted hazard ratio for weekly change [aHR] 1.08 (1.03, 1.14), earlier discontinuation of mechanical ventilation in Chittagong (aHR 2.97 (1.24, 7.14), p = 0.02), and earlier ICU discharge in Patan (aHR 1.87 (1.02, 3.43), p = 0.04).

**Conclusion:**

This structured training program was associated with a decrease in ICU mortality in two of three sites and improvement of other performance indicators. A larger cluster randomised study assessing process outcomes and longer-term indicators is warranted.

## Introduction

Although access remains limited, intensive care (ICU) facilities are increasingly available in many low and middle income countries.[1–6] Nonetheless, from the limited data available, case fatality rates in these ICUs remain high.[7,8] A lack of both material resources and trained staff is likely to be an important contributor.[9] But within these limits better use of available ICU resources and adherence to basic principles of critical care have the potential to reduce mortality without increasing cost of treatment. These principles, however, will have to be translated into setting-specific strategies, adapted to the local infrastructure and disease case mix.[10] Quality training in critical care medicine is a prerequisite for good care of the ICU patient, but formal training programs are either absent or minimal in low-income and sometimes also in middle income countries.[3,5,6] The importance of training for medical and nursing staff is being increasingly emphasized by those seeking to improve ICU performance and increase capacity in these settings.[9,11]

An international critical care training collaboration with the aim to improve patient outcomes within the constraints inherent to the resource-limited setting was convened. It was hypothesised that the introduction of a modular training program would lead to improvement in ICU performance indicators: with a reduction in the disease severity–adjusted mortality, length of ICU stay, duration of mechanical ventilation, duration of vasoactive drug use and in the duration of antibiotic therapy.

## Methods

The modular training programme, with a focus on practical bedside teaching, aimed at critical care physicians and nurses in three ICUs in, Rourkela, India (11–bed ICU at Ispat General Hospital), Chittagong, Bangladesh (9–bed ICU at Chittagong Medical College Hospital) and Patan, Nepal (6–bed ICU at Patan Hospital) was instituted. These hospitals were chosen for pragmatic reasons as they were part of existing research collaborations between participating institutions and were willing to participate in the project. Teachers consisted of ICU doctors and nurses trained in Europe or the U.S.A (see collaborators, below). This multifaceted training programme evaluated using a two-phase interrupted times series design at three sites. ICU routine performance indicators were collected over a period of three years: from 8 to 15 months before the start of the intervention (pre-intervention period) until 18–24 months after (post-intervention period) for evaluation of the programme. The training was focused on the basic tenets of intensive care and was analogous to the continuing professional training for doctors and nurses carried out in most ICUs in high income countries. No direct patient interventions were performed and no additional tests or new data collection implemented. Approval for the programme was obtained from the relevant units and institutions, and a waiver for ethical approval including waiver of consent, was obtained from the Oxford Tropical medicine Research Ethical Committee (OXTREC).

### Setting

The participating ICUs all had facilities for ventilation but not for bedside haemodialysis. Peritoneal dialysis and intermittent out-of-ICU haemodialysis was available in all three hospitals. Multi-parameter electronic patient monitoring including non-invasive blood pressure monitoring was present in all three units along with infusion pumps for drug administration, though resources were often limited, and equipment shared between patients.

ICU nurses staffed the three units with 1: 1 to 1:2 nurse-to-patient ratios during the day and a minimum ratio of 1:3 at night. The doctors in charge of the ICUs were primarily anaesthetists in Rourkela and Chittagong, with internal medicine specialists in charge of the unit in Patan. Junior medical staffing, similarly, was predominantly by trainee anaesthetic doctors in Rourkela and Chittagong with internal medicine trainees in Patan. All ICUs had round the clock coverage of doctors.

The ICU and hospital in Rourkela, a steel–mining town, is funded by the steel company employing a large proportion of the town’s population. Employees of the company and their relatives enjoy free medical care at the ICU while others were required to pay. The ICU in Chittagong was publicly funded (though patients paid a subsidised daily fee for occupancy and for disposables/drugs when these were not available in the hospital). In Patan the ICU and hospital were based on a “fee for service” model. In all three sites, but to varying extents, a “poor patient” fund was in place to provide limited support for those unable to afford care.

### Intervention

The intervention consisted of a six-module training programme on basic intensive care practices over a period of 20 months evaluated by performance indicators before, during and after the training programme.

Each module consisted of two to three weeks of short classroom lectures in the morning complemented by extensive bedside teaching for both doctors and nurses during the rest of the day. A brief needs-analysis was performed by the training team at the commencement of the training model; this was then used to guide the delivery of the training module. Each of the 18 training teams consisted of an experienced intensivist and an ICU nurse (see supplement 1 for list of trainers) and trained approximately all 100 doctors and nurses across the three sites (30 each in Patan and Chittagong and 40 in Rourkela). The modules covered the following themes: i) Basic care for the critically ill patient; ii) Care on admission and in emergency situations; iii) Shock and its treatment; iv) Feeding, glucose control and monitoring; v) Care beyond the initial phase; vi) Remaining issues, which depended on local demand and remaining observed deficiencies. When appropriate, local staff in collaboration with the trainers were encouraged to produce locally adapted treatment guidelines, for instance on severe sepsis management (see supplement 1 for further details of the training program). The training was conducted in English, with on–site translation for nurses in Bangladesh.

### Outcomes

To assess the training impact we collected (from routine patient records) a range of demographic, clinical and resource utilization data on patients admitted to the ICUs before, during and after the implementation of the training modules. The intervention was non-randomized and the analysis focused on describing the change in pre-defined indicators prior to and after the commencement of the training program. These data were extracted from routine patient records of patients admitted to the ICU during the following periods: for Rourkela—every second patient (due to high case load and limited availability of human resources) admitted between August 2009 to June 2012; for Chittagong all patients admitted between June 2009 to June 2012; and for Patan between February 2009 and December 2011 (limited by logistics of data extraction). Clinical data included diagnosis at time of ICU admission, comorbidities, physiology at time of admission and organ support initiated on arrival to ICU. Physiology and laboratory parameters during the first 24 hours needed to calculate APACHE II scores (the simplest of the commonly used critical care prognostic scoring systems) were also extracted. Discharge information consisted of ICU survival, durations of mechanical ventilation, vasoactive drugs and antibiotic treatment. The study did not attempt to collect hospital or 28-day outcomes due to infeasibility of such follow-up in these settings. The study did not capture information regarding readmissions due to the lack of unique identifiers during repeat admissions in the three hospitals.

All data extracted were stored in the ICUs, entered into a password protected electronic database and then securely disposed of. Data extraction was performed by trained nurses in Rourkela while in Chittagong and Patan it was performed by trained medical officers working in the hospitals.

### Statistical analysis

We assessed the effect of the intervention on three process outcomes and three patient outcomes. Process outcomes were time on mechanical ventilation, time to discontinue vasoactive drugs or to stopping antibiotics (amongst those ventilated, taking vasoactive drugs or antibiotics within 24 hours of ICU admission respectively). Patient outcomes were cumulative incidence of ICU mortality, ICU mortality rate, and the rate at which patients were discharged alive from the ICU. Analyses were performed separately for each of the three centers. ICU mortality and discharge from the ICU alive were considered competing events. To account for this the effect of the intervention on the cumulative incidence of ICU mortality was assessed with a Fine and Gray model.[12] This accounts for the possibility that the intervention may change both the mortality rate and patient discharge rate; both will affect the cumulative incidence of ICU mortality.[13] In this case the association between the intervention and outcome is reported with the adjusted sub-distribution hazard ratio (aSHR). An aSHR less (greater) than one indicates an association with a reduced (increased) *risk* of the outcome. For other outcomes we estimated cause-specific hazards using a Cox proportional hazards model, with censoring at the time of the competing events (death or discharge). In this case the association between the intervention and outcome is reported with the adjusted hazard ratio (aHR), An aHR less (greater) than one indicates an association with a reduced (increased) *rate* of the outcome, where the rate is the risk (i.e. probability) of the event per unit time. Models also allowed for pre-training trends. In all models we allowed for both an immediate step change due to the start of the intervention and a change in trend following the intervention to account for a possible learning curve associated with the intervention. Trends were assumed to be linear on the log hazard scale. Models adjusted for sex, age, season (rainy season or otherwise), type of admission (medical or otherwise), and APACHE II probability, unless there was evidence to reject the proportional hazards assumption in which case stratification was used. This was the case for ICU mortality rate in Chittagong (stratified by whether or not admission was classified as “medical”) and antibiotic stopping rate at Rourkela (stratified by medical and sex)

Outcomes from the Fine and Gray model are expressed through the sub-distribution hazard ratio while results of the Cox models were expressed through the hazard ratio (values greater than one indicate an association with, respectively, an increased risk or rate of the outcome). Analysis was performed using STATA 13 (College Station, Texas) according to the pre-specified analysis plan.

## Results

A total of 3,868 patients were included in the dataset, with over two–thirds of these admitted to the Rourkela ICU. The median age was 52 years in Rourkela, 32 in Chittagong and 54 in Patan. Further demographic, clinical and severity of illness information along with ICU discharge status pre and post-intervention are summarised in Table 1.

**Table 1:** Demography, clinical profile, severity of illness and ICU discharge status of patients in the three ICUs admitted during the before and after commencement of training period.

	Pre-intervention			Post-intervention		
Site	Rourkela	Chittagong	Patan	Rourkela	Chittagong	Patan
**Patients (n)**	822	103	122	1777	616	293
**Age, y (mean, range)**	49.7(0 .5–95)	39.7(7–96)	54.3(9–95)	49.6(1.5–95)	36.6(10days-98)	49.8(12–90)
**% Male**	69.80%	65.40%	50%	68.90%	49.50%	44.40%
**APACHE II(mean, SD)**	24.7(7.5)	13.8(6.4)	9.2(5.9)	24.5(7.9)	12.7(5.9)	9(5.7)
APACHE II pr*(mean, SD)	0.125(0.13)	0.162(0.146)	0.121(0.102)	0.124(0.125)	0.182(0.152)	0.123 (0.112)
**Dead at ICU discharge (%)**	228(28%)	64(62%)	50(41%)	525(30%)	313(51%)	53(18%)

* probability

**Fig 1:**
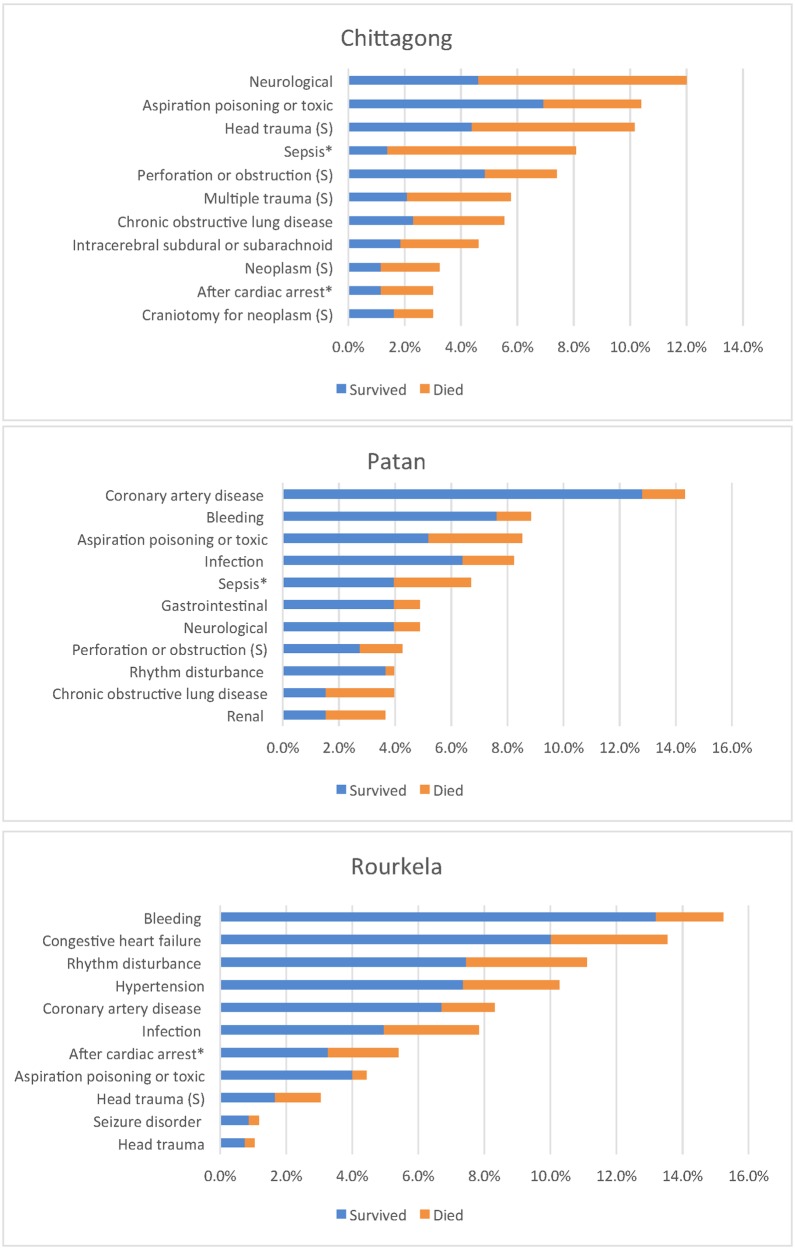
Common admission diagnoses (APACHE II diagnostic category) and mortality by site during study period.

The frequency of the most common diagnoses and their associated mortality for each site are shown in Fig 1. ICU mortality in the pre and post intervention periods respectively were as follows Rourkela 28% and 30%, Chittagong 62% and 51% and Patan 41% and 18%.

**Fig 2:**
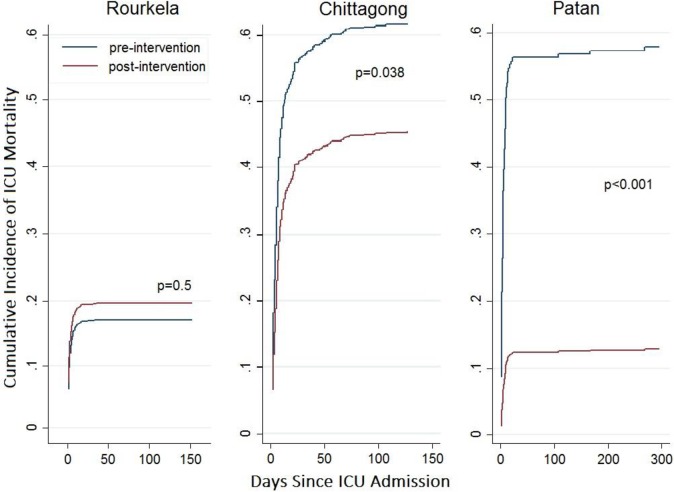
Cumulative incidence function for ICU mortality in the three study sites. P-values refer to the evidence for stepwise changes in adjusted subdistribution hazard ratio from the Fine-Gray model for the cumulative incidence of ICU mortality.

**Fig 3:**
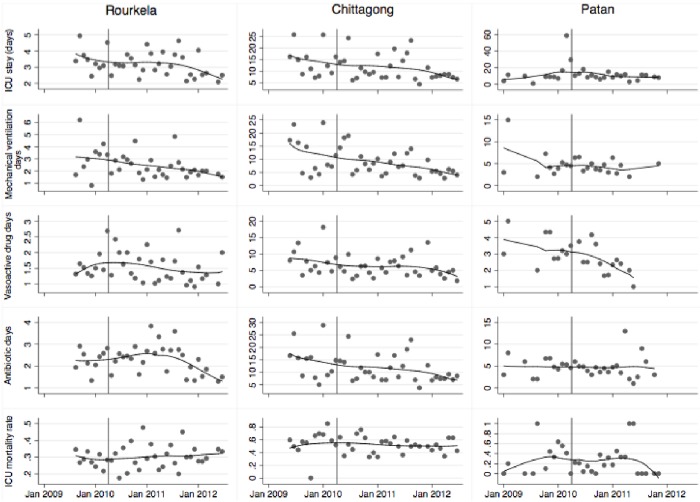
Median duration of ICU stay, mechanical ventilation, vasoactive drug use and antibiotic use in the three study sites by four-week period. The trend line is a non-parametric locally weighted scatterplot smoother. The vertical line indicates the time that the training intervention started.

The cumulative incidence of ICU mortality between pre-training and post-periods was associated with a substantial stepwise reduction at Chittagong and Patan as indicated by a Phase 2 step change aSHR of less than one (Fig 2, Table 2). There was no evidence at Rourkela that the intervention resulted in reduced ICU mortality. At Chittagong, the Phase 1 trend aSHR was above one 1 (95% confidence interval [1.00, 1.04]), providing evidence of a tendency for cumulative incidence of ICU mortality to increase over calendar time during the pre-intervention period; the intervention was associated with a reversal of this trend reflecting a post-intervention reduction in the ICU mortality rate and increase in the ICU discharge rate (Table 2). The steep increase in the cumulative incidence of ICU mortality in the first few weeks following admission at all three centers and subsequent levelling off reflects the short length of ICU stays (Fig 3). Since very few patients remain in ICU for more than 50 days, the cumulative incidence of ICU mortality changes little after this time period.

**Table 2:** Trends for outcomes in the three study sites.

**Rourkela**	**Outcome**					
**Variable**	Cumulative incidence of ICU mortality aSHR (95% CI)^1^	ICU mortality rate aHR (95% CI)^2^	ICU discharge alive rate aHR (95% CI)^2^	Rate of stopping mechanical ventilation aHR (95% CI)^2^	Rate of stopping vasoactive drugs aHR (95% CI)^2^	Rate of stopping antibiotics aHR (95% CI)^2^
**Phase 1 trend**^3^	0.99(0.98–1.01),p = 0.55	1.01(0.99–1.03),p = 0.55	1.01(1.00–1.02),p = 0.07	1(0.97–1.04),p = 1.00	0.93(0.880.98),p = 0.01	0.99(0.96–1.01),p = 0.30
**Phase 2 step change**	1.17(0.75–1.82),p = 0.49	0.96(0.61,1.51),p = 0.87	0.9(0.70–1.15),p = 0.40	0.94(0.45–1.93),p = 0.86	2.45(0.76–7.88),p = 0.13	0.56(0.32–1.00),p = 0.05
**Phase 2 change in trend**^3^	1.01(0.99–1.03),p = 0.50	1(0.98–1.02),p = 0.68	0.99(0.98–1.00),p = 0.06	1.01(0.97–1.04),p = 0.69	1.08(1.03–1.14),p = 0.002	0.99(0.98–1.01),p = 0.55
**Chittagong**	**Outcome**					
**Variable**	Cumulative incidence of ICU mortality aSHR (95% CI)^1^	ICU mortality rate aHR (95% CI)^2^	ICU discharge alive rate aHR (95% CI)^2^	Rate of stopping mechanical ventilation aHR (95% CI)^2^	Rate of stopping vasoactive drugs aHR (95% CI)^2^	Rate of stopping antibiotics aHR (95% CI)^2^
**Phase 1 trend**^3^	1.02(1.00–1.04),p = 0.03	1.01(0.99–1.03),p = 0.51	0.99(0.97–1.02),p = 0.47	0.96(0.93–1.00),p = 0.03	0.99(0.95–1.04),p = 0.77	0.98(0.92–1.04),p = 0.49
**Phase 2 step change**	0.62(0.40–0.97),p = 0.04	0.9(0.54–1.49),p = 0.69	1.6(0.85–3.02),p = 0.15	2.97(1.24–7.14),p = 0.02	1.2(0.43–3.35),p = 0.72	1.26(0.27–5.91),p = 0.77
**Phase 2 change in trend**^3^	0.98(0.96–1.00),p = 0.03	0.99(0.97–1.01),p = 0.55	1.01(0.99–1.04),p = 0.34	1.04(1.01–1.08),p = 0.01	1.01(0.97–1.06),p = 0.59	1.04(0.98–1.10),p = 0.23
**Patan**	**Outcome**					
**Variable**	Cumulative incidence of ICU mortality aSHR (95% CI)^1^	ICU mortality rate aHR (95% CI)^2^	ICU discharge alive rate aHR (95% CI)^2^	Rate of stopping mechanical ventilation aHR (95% CI)^2^	Rate of stopping vasoactive drugs aHR (95% CI)^2^	Rate of stopping antibiotics aHR (95% CI)^2^
**Phase 1 trend**^3^	1.02(1.00–1.05),p = 0.08	1.01(0.99–1.04),p = 0.38	0.99(0.97–1.00),p = 0.11	1(0.97–1.02),p = 0.90	1.01(0.97–1.06),p = 0.60	1.01(0.98–1.04),p = 0.42
**Phase 2 step change**	0.16(0.06–0.41),p<0.001	0.22(0.08–0.62),p = 0.004	1.87(1.02–3.43),p = 0.04	1.95(0.51–7.4),p = 0.33	2.04(0.45–9.16),p = 0.36	1.85(0.82–4.18)p = 0.14
**Phase 2 change in trend**^3^	1(0.96–1.03),p = 0.82	1.01(0.97–1.04),p = 0.69	1.01(0.99–1.03),p = 0.23	0.99(0.95–1.03),p = 0.70	0.98(0.93–1.04),p = 0.57	0.98(0.95–1.01),p = 0.24

aHR, adjusted hazard ratio; aSHR, adjusted sub-distribution hazard ratio

^1^ Results are based on the competing risks regression, which accounts for the effect of the intervention. aSHR is the adjusted sub-distribution hazard ratio, adjusting for sex, age, reason, APACHE II probability, and whether the patient is a medical admission.

^2^ Results are based on a Cox regression model

^3^ Time units are expressed in weeks, so reported aSHR and aHR represent proportional change in outcome each week

Length of ICU stay, duration of mechanical ventilation, vasoactive drug days and antibiotic days all demonstrated reductions in the post-intervention period at Rourkela and Chittagong and vasoactive drug days decreased at Patan (Fig 3). Accounting for possible confounding factors (continuation of pre-intervention trends, changes in mortality and ICU stay, changes in patient mix, changes in bed occupancy, and changes in the number of patients starting mechanical ventilation, vasoactive agents or antibiotics) in the survival analysis indicated that the training was associated with earlier discontinuation of vasoactive drugs at Rourkela, shorter time on mechanical ventilation at Chittagong, and faster discharge from ICU at Patan (Table 2). There was, however, no evidence that the intervention was associated with an increase in the rate of stopping antibiotics at any site, and some evidence of the converse effect at Rourkela.

## Discussion

The structured critical care training programme was associated with a reduction in ICU mortality in two of the three study sites with a positive effect on at least one of the predefined process outcome indicators at each site, including duration of mechanical ventilation or vasoactive drug use and the time to ICU discharge (Table 2). There was no significant impact on the duration of antibiotic use. Study sites differed considerably in case-mix, staffing and staff workload, resources, management and treatment practices. The positive effects on case fatality and other impact indicators were independent of the differences in severity of disease and other variables between sites and over time.

The larger reductions in mortality and lengths of stay in Chittagong could be related to more successful implementation of the training (for example by the institution of a twice daily inter-professional ICU ward round [14]) but can also be explained by high baseline case fatality compared to the other two sites. The absence of any effects on mortality in Rourkela could be due to the short ICU stay and high turnover of patients. The relatively small patient numbers in the pre-intervention phase should be noted when interpreting the relatively high baseline mortality during that phase in Patan and Chittagong. The reduction in duration of mechanical ventilation was accompanied by a larger proportion of patients being mechanically ventilated; this relationship was most noticeable in Rourkela (S2 Fig).

There is increasing awareness of the importance of identifying strategies capable of improving intensive care in resource limited settings.[11,15] Our study highlights the importance of training aiming at both doctors and nurses for improving ICU performance. The potentially vast gains achievable by focusing on improving the knowledge, skills and attitudes of ICU staff in resource limited environments is remarkable and should stimulate the implementation of similar local capacity building measures. It further demonstrates the applicability and benefit of basic intensive care good clinical practice across these three sites even when resources and workload are obvious challenges. The gradual improvement, and the persistence of the changes after the last module, suggests a sustained change in outcomes related to staff knowledge and skills, stimulated by the trainers and the training program. Longer-term sustainability of these changes is worthy of future investigation.

In high-income countries, implementation of so–called ‘care bundles’ for the treatment of severe sepsis have been shown to improve outcome in ICUs. The effects of training programs in resource-limited settings are rarely evaluated in terms of results or outcome improvements (Kirkpatrick model—level 4,[16]) and the limited reports available focus on protocol implementation related to sepsis management,[17] infection prevention,[18] or the teaching of specific clinical skills.[19] In contrast, our training program addressed a wide spectrum related to the basic aspects of critical care practices and demonstrated improvement across diverse predefined performance indicators in ICUs in three countries.

It became apparent during our program that in addition to specific ICU technical aspects of care, organisational and basic nursing care aspects were equally important to address. This included the need for patient-centred nursing, empowerment of nurses on treatment decisions in acute situations, the need to improve attitudes with regard to teamwork, monitoring of vital signs with proper alarm limits settings prompting appropriate action, use of both short- and long-term treatment plans, and accurate patient documentation with systematic hand-overs for the new shift. Enforceable admission and discharge policies, consistent and structured staffing rotas for doctors and nurses with adequate supervision, development of governance and audit policies, guidelines on accepting and transferring patients to and from other institutions were additional areas for improvement. The training program was deliberately not rigorously controlled for content or format and the training teams were encouraged to address these additional aspects and prioritise content as per local need and learning capability.

The structure of short classroom-type presentations for theoretical knowledge, but with an emphasis on bedside training in core critical care skills proved an appropriate teaching model and can be a template for future training programs. The combined teaching model enabled training to be conducted in each three week period catering to the highest possible number of staff whilst minimising disruption to patient care in these ICUs. The training program can also contribute to the development of a portfolio of critical care training material. However, high travel costs and the limited number of units and personnel who can be reached at any one time are barriers for scaling up the presented model. A more sustainable and scalable program should focus on creating a critical mass of local trainers within these countries and across the region to initiate a “train the trainer” model.[20] Mentoring trainees by utilising the increasing local critical care specialists, for example in India, can facilitate such programs. A shortage of trainers might be addressed temporarily by using other training methods including a manual, online training, audio-visual material reinforced with limited face-to-face teaching. This more regimented model which requires less time of face-to-face teaching is more often used internationally.[21] However, its effectiveness in resource-poor settings will need to be assessed. Another option is visits from selected staff to ICUs in high-income countries, providing exposure to state of the art intensive care practices that can serve as a reference standard. However, in our experience the benefits of such visits are generally limited because of the vast disparities in the organisational, governance, equipment, patient case-mix and language elements between the two settings.

The training program was highly appreciated and an important learning experience for both the trainers and recipients. A focus of the training course was on implementation of evidence based interventions in a setting-relevant manner. One of the early outputs was a setting-adjusted guideline for the treatment of severe sepsis designed by the local teams in consultation with the training teams (S3 Fig). This exercise emphasized the need for re-evaluation of many aspects of the ‘surviving sepsis’ guidelines in resource poor settings. Not only are many of the recommendations not fully implementable because of the material limitations, but also the different local patient case-mix can prompt additional adaptation of the guidelines. An example is modified fluid management guidelines required in severe sepsis syndromes caused by dengue or malaria.

In our project, we encountered difficulties in data extraction from paper records (especially in Patan), even when attempted during the patient’s stay in the ICU. We suspect that this will be a common theme in similar settings where electronic records and continuous surveillance or registry processes are absent. Implementation of setting adjusted electronic ICU registries will be an important way forward to address this issue. This will also greatly facilitate future evaluation of training interventions, in which evaluation of impact on outcomes is essential.

A limitation of our study is that the quasi-experimental nature of the design precludes a strong causal interpretation; post-intervention changes in outcomes may reflect changes in patient characteristics or other aspects of care unrelated to the intervention. While adjustment for patient-level covariates (including APACHE II probabilities) goes some way to reducing this risk, the study remains vulnerable to presence of important unmeasured confounders. It is also possible that incomplete sampling of patients in Patan led to bias. Nevertheless, results are encouraging enough to suggest a larger cluster randomized study able to provide more definitive evidence could be warranted. Ideally such a study should be designed to detect changes in an endpoint of unambiguous clinical relevance such as 28-day mortality which due to resource constraints was not possible here and which are not routinely available in such settings.

A further shortcoming of our project was that we did not evaluate the knowledge and skills of staff pre and post training and subsequent changes in level of implementation of these skills. We also did not control for the impact of staff turnover and did not explore barriers to implementation of already existing knowledge and skills. Furthermore we did not monitor the extent to which the training was adhered to and whether the levels achieved post-intervention were maintained subsequent to the departure of the trainers. Our study was not designed for the evaluation of individual modules therefore conclusions with regard to the impact of specific areas of the training could not be made. Data availability posed additional limitations, especially in Patan. APACHE II may not be predictive of mortality in a low-middle income setting, given differences in patient characteristics. Further we did not record hospital, 30 or 90-day survival data.

## Conclusion

This broad, pragmatic multidisciplinary training program, performed in three diverse resource limited ICUs was associated with a decrease in ICU mortality and improvement of other performance indicators. Providing setting-adapted training is important to improve ICU performance in resource-limited settings.

## Supporting information

S1 Fig Adapted sepsis guidelines prepared by Rourkela ICU team based on training module on sepsis. (PDF)

S2 Fig Patient costs for drugs and fluids in Rourkela and Chittagong (data for Patan were not available). (PDF)

S3 Fig The relationship between duration of mechanical ventilation and proportion of patients being mechanically ventilated in Rourkela. (PDF)
