# Supplementary material for: Impact of a structured ICU training programme in resource-limited settings in Asia
Source: PLoS One. 2017 Mar 14;12(3):e0173483. doi: 10.1371/journal.pone.0173483 (PMC5349661; doi:10.1371/journal.pone.0173483)
Supplement: S1 Fig — (PDF) [file pone.0173483.s001.pdf]

Supporting Information (S1)

Supplement 1 – Description of training program

The training program across all three sites consisting of 6 modules commenced in April 2010 and continued till January 2012 as indicated in table 1, below.

Table 1 : Timeline of the training program in Rourkela, Chittagong and Patan

Module	Time	Rourkela	Chittagong	Patan
1	**April/May 2010**	Basic care for critically ill		
2	**September 2010**	Care on admissions and emergencies	Care beyond the initial phase	Shock and its treatmenet
3	**January 2011**	Shock and its treatmenet	Care on admissions and emergencies	Feeding, glucose control and monitoring
4	**May 2011**	Feeding, glucose control and monitoring	Shock and its treatmenet	Care beyond the initial phase
5	**September 2011**	Care beyond the initial phase	Feeding, glucose control and monitoring	Care on admissions and emergencies
6	**January 2012**	Recap of above modules and tackling of any remaining issues		

Each module was led by an experienced intensivist and nurse from the UK, US, Belgium and the Netherlands. The list of trainers and their affiliations is listed at the end of this supplement. Each module lasted between two and three weeks with the trainers being on site and working as a team throughout this period.

The material and methods used for each training module were not tightly controlled. The trainers were provided with the main components of each module and allowed shared use of the material developed by other trainers. The main components of each teaching module the training team were requested to focus were as follows:

Module 1- basic care for critically ill

- ward rounds and handover for doctors and nurses
- airway management and mechanical ventilation

- source control and treatment of infection

Module 2- Care on admissions and emergencies

- admission protocols and policies
- teamwork in managing emergency situations
- fluid management and resuscitation

Module 3- Care beyond the initial (admission) phase

- use of sedation and paralysis
- care of the unconscious critically ill patient
- pain and delirium management

Module 4- Shock and its treatment

- cardiovascular monitoring of the shock patient
- use of inotropes and vasopressor drugs
- reassessment and targets for resuscitation

Module 5- Feeding, glucose control and monitoring (use of tests/imaging)

- feeding – timing, route and benefits
- glucose control
- Use of tests and imaging for monitoring critically ill patient

Module 6- Recap of above modules and tackling any remaining issues

A stepwise approach to implementation of therapy or strategies was utlised as follows for each module:

- **A brief situational analysis and environmental scan focusing on the therapy or strategy of interest** – assessment of current practice and knowledge regarding the specific therapy or strategy, and identification of benefits or risks

- **Identification of fears, barriers and facilitators for the therapy or strategy of interest** – fears and barriers identified and rationalized; facilitators identified who can be used to further implementation process
- **Development of local guidelines on the therapy or strategy of interest** – appropriate guidelines developed in collaboration with the local team and reviewed several times until all caregivers involved in the performance understand all parts
- **Application of the guidelines on the therapy or strategy of interest** – encourage use of the guidelines guided by peers, who were involved in the development of the guidelines

When appropriate, as in the treatment of shock, local staff in collaboration with the trainers were encouraged to produce locally adapted treatment guidelines, for instance on severe sepsis management (see supplement 2, below).

The following is an example of topics addressed at the bedside by one team to train the local doctors and nurses on mechanical ventilation:

- recognize respiratory failure
- determine the cause of respiratory failure
- recognition of effective and ineffective ventilation
- initiate mechanical ventilation by setting the ventilator
- clearing the airway and bag–squeezing
- guiding mechanical ventilation – management of CO2 removal (minute ventilation), management of oxygenation (PEEP, recruitment), timing of change form mandatory to spontaneous breathing
- understand the role of mechanical ventilation in lung injury
- lung protective mechanical ventilation and recognition of associated complications
- recognize problems with mechanical ventilation through airway pressure tracings
- monitoring a patient during weaning and interpretation of weaning parameters

A breakdown of a typical training day is as follows:

0900: recap of previous days training

0915-1000: septic shock – introduction and pathophysiology

1000-1100: septic shock-treatment goals and barriers

1130-1230: local septic shock guidelines – group work: plan for design

1330-1630: bedside teaching on patients with sepsis and septic shock to highlight learning goals from the day

1630-1700: debrief, summary and plan for next day

The list of trainers (alphabetically) with their affiliations, who delivered training in the three sites (some delivered training in more than one site): Wim van den Berg, MD Department of Critical Care University Medical Center Groningen, The Netherlands Helga Birkhoff, ICU RN ICU Academic Medical Center, Amsterdam, The Netherlands: Rebecca Boonman, ICU RN ICU Academic Medical Center, Amsterdam, The Netherlands; Andreas Brunauer, MD Anesthesiology and Intensive Care, Landeskliniken Salzburg, Austria; , Evelyne Desmet, ICU RN ICU Academic Medical Center, Amsterdam, The Netherlands; Roelie Deuten, ICU RN ICU Academic Medical Center, Amsterdam, The Netherlands; Arjen Dondorp MD, University of Oxford, UK; Frank Dullemeijer, ICU RN ICU Academic Medical Center, Amsterdam, The Netherlands;, Henrik Endeman, MD Department of Intensive Care, Onze Lieve Vrouwe Gasthuis, Amsterdam, The Netherlands; Timothy McErlane, MD ICU Royal Berkshire Hospital, UK; Siebe van der Geest ICU Maastricht University Medical Center, The Netherlands; Rashan Haniffa, MD University of Oxford; Janneke Horn, MD ICU Academic Medical Center, Amsterdam, The Netherlands; Nicole Juffermans, MD ICU Academic Medical Center, Amsterdam, The Netherlands; Wendy van de Laar, ICU RN ICU Academic Medical Center, Amsterdam, The Netherlands; Erik-Jan van Lieshout, MD ICU Academic Medical Center, Amsterdam, The Netherlands; Elly Nederstigt ICU RN ICU Academic Medical Center, Amsterdam, The Netherlands;, Anne-Cornelie de Pont, MD Academic Medical Center, Amsterdam, The Netherlands; Ton van Rijn, ICU RN ICU Academic Medical Center, Amsterdam, The Netherlands; Louise van

Rossum, ICU RN ICU Academic Medical Center, Amsterdam, The Netherlands; Krishna Sadhoe, ICU RN ICU Academic Medical Center, Amsterdam, The Netherlands; Miet Schetz, MD Department of Intensive Care, University Hospital Leuven, Belgium; Marcus Schultz, MD Academic Medical Center, Amsterdam, The Netherlands; Lesley van Sonsbeek, ICU RN ICU Academic Medical Center, Amsterdam, The Netherlands; Peter Spronk, MD ICU, Gelre Hospitals Apeldoorn, The Netherlands; Lucia Stena, ICU RN ICU Academic Medical Center, Amsterdam, The Netherlands; Nancy Sterkenburg, ICU RN ICU Academic Medical Center, Amsterdam, The Netherlands; Rianne Verhoef, ICU RN ICU Academic Medical Center, Amsterdam, The Netherlands; Andy Walden, MD ICU Royal Berkshire Hospital, UK; Ducky de Vries, ICU RN ICU Academic Medical Center, Amsterdam, The Netherlands; Eoin West, MD Harborview Medical Center, University of Washington, Seattle, USA, Tineke Winters, ICU RN ICU Academic Medical Center, Amsterdam, The Netherlands; Tineke Zwiers, ICU RN ICU Academic Medical Center, Amsterdam, The Netherlands.
