# Supplementary material for: Impact of a structured ICU training programme in resource-limited settings in Asia
Source: PLoS One. 2017 Mar 14;12(3):e0173483. doi: 10.1371/journal.pone.0173483 (PMC5349661; doi:10.1371/journal.pone.0173483)

Supporting Information (S2 Fig)

Supplement 2: Adapted sepsis guidelines prepared by Rourkela ICU team based on training module on sepsis

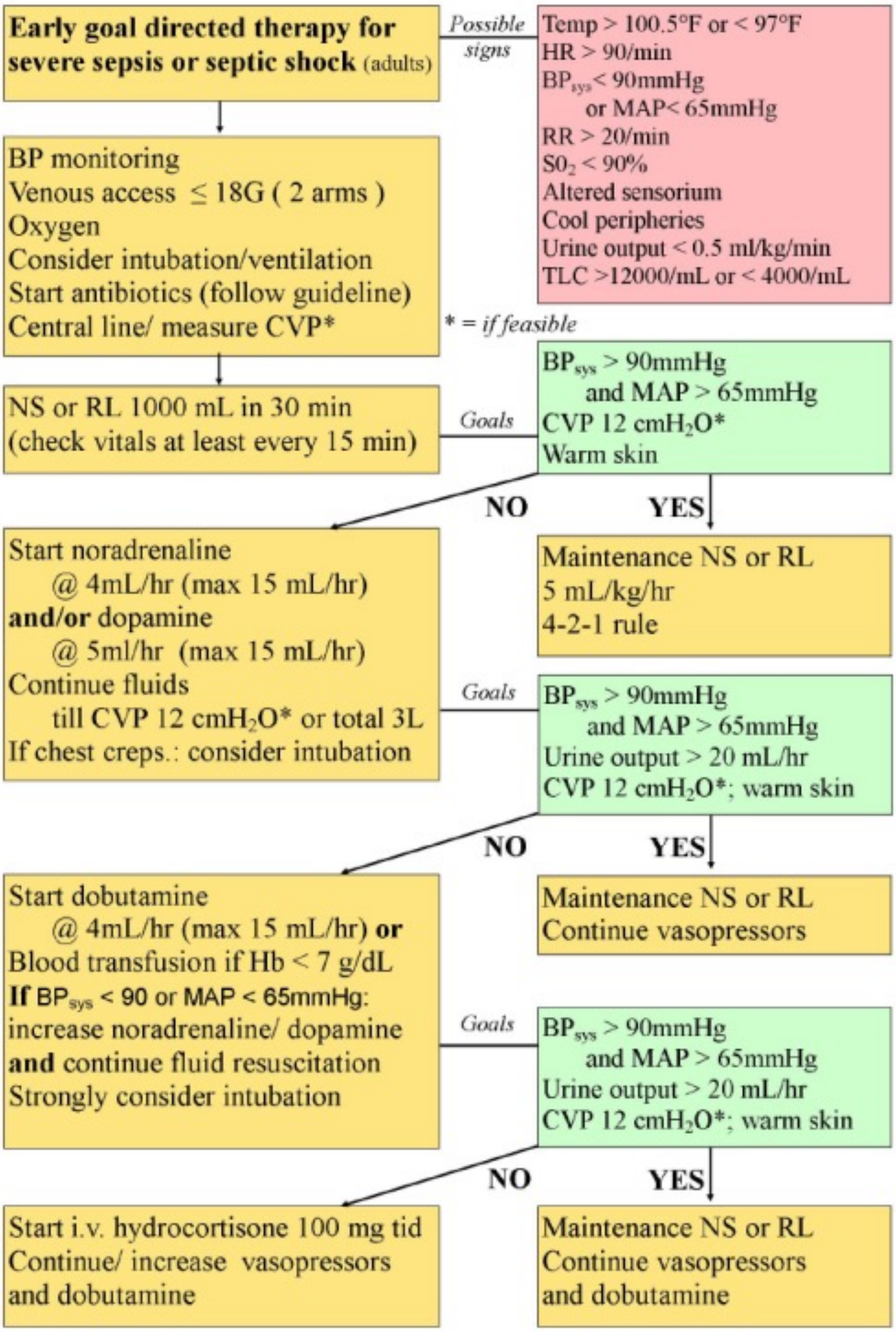

Supplement: S2 Fig — (PDF) [file pone.0173483.s002.pdf]
