# Supplementary material for: Impact of a structured ICU training programme in resource-limited settings in Asia
Source: PLoS One. 2017 Mar 14;12(3):e0173483. doi: 10.1371/journal.pone.0173483 (PMC5349661; doi:10.1371/journal.pone.0173483)

Supporting Information (S3 Fig)

Supplement 3: The relationship between duration of mechanical ventilation and proportion of patients being mechanically ventilated in Rourkela

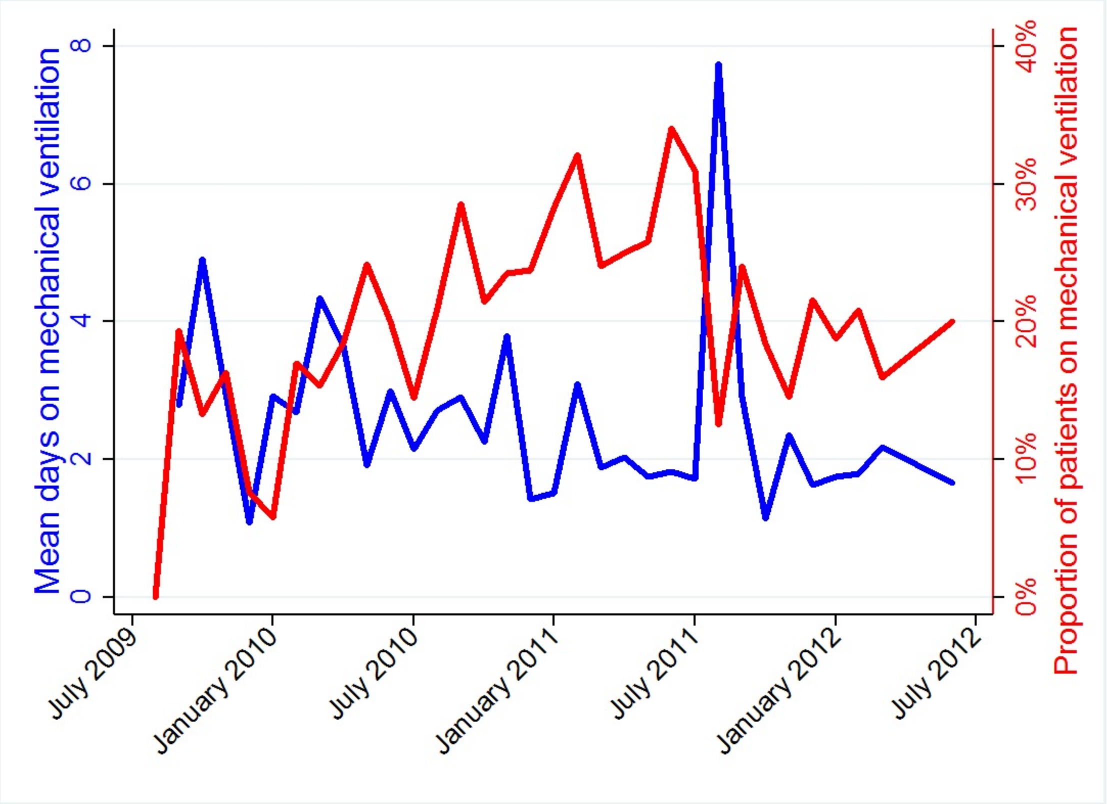

Supplement: S3 Fig — (PDF) [file pone.0173483.s003.pdf]
